# Time trends in emotional well-being and self-esteem in children and adolescents during the COVID-19 pandemic

**DOI:** 10.1186/s13034-022-00525-3

**Published:** 2022-11-23

**Authors:** Ryunosuke Goto, Aurelie Piedvache, Mayumi Hangai, Yui Yamaoka, Mariko Sampei, Naomi Sawada, Yusuke Okubo, Kyoko Tanaka, Naho Morisaki, Mariko Hosozawa

**Affiliations:** 1 Department of Pediatrics, The University of Tokyo Hospital, Tokyo, Japan; 2 Department of Social Medicine, National Center for Child Health and Development, Tokyo, Japan; 3 Department of Pediatrics, Graduate School of Medicine, The University of Tokyo, Tokyo, Japan; 4 Department of Global Health Promotion, Tokyo Medical and Dental University, Tokyo, Japan; 5 Department of Health Communication, Graduate School of Medicine, The University of Tokyo, Tokyo, Japan; 6 Department of Psychosocial Medicine, National Center for Child Health and Development, Tokyo, Japan; 7 Bureau of International Health Cooperation, National Center for Global Health and Medicine, Institute for Global Health Policy Research, Tokyo, Japan

**Keywords:** COVID-19, Social distancing, School closures, Emotional well-being, Self-esteem

## Abstract

**Objective:**

Given their unique COVID-19 pandemic experience, it is necessary to evaluate the mental health of youth beyond the initial stages of the pandemic, in relation to the stringency of the social distancing measures. We aimed to describe long-term trends in emotional well-being and self-esteem among youth in Japan during the pandemic.

**Method:**

Using serial cross-sectional data from April 2020 to December 2021, we evaluated the trends in emotional well-being and self-esteem of youth aged 6–17 years using the self-report KINDL questionnaire, weighted to represent the age and gender distributions in the Japanese population. We then tested the associations between emotional well-being and self-esteem and stringency of social distancing policies, measured using the Oxford COVID-19 Stringency Index. Analyses were also stratified by gender and age group.

**Results:**

The emotional well-being and self-esteem of youth improved transiently in 2020, followed by a slight worsening trend into 2021. While emotional well-being stayed lower compared to initial levels nearly 2 years into the pandemic, self-esteem began to improve by late 2021. 12–17 year-olds had lower emotional well-being and self-esteem compared to 6–11 year-olds throughout the study period. Females had lower emotional well-being than males in May 2020 and lower self-esteem than males in May and September/October 2020. More stringent social distancing measures were associated with lower emotional well-being and self-esteem, especially 6–11 year-olds’ self-esteem and females’ emotional well-being.

**Conclusion:**

During the COVID-19 pandemic, older youth tended to have lower emotional well-being and self-esteem than younger youth. Younger and female youth were especially vulnerable to stringent social distancing measures.

**Supplementary Information:**

The online version contains supplementary material available at 10.1186/s13034-022-00525-3.

## Background

The COVID-19 pandemic has presented unprecedented challenges to the mental health of children and adolescents. Recent studies have revealed that mental illnesses among children and adolescents increased during the pandemic [1–3]. For instance, Newlove-Delgado and colleagues showed that in July 2020, 5- to 16-year-olds in England experienced increased mental health problems compared to before the pandemic [2]. While such studies provide valuable evidence amidst an unprecedented crisis, most studies were conducted during the initial stages of the pandemic (i.e., up to early 2021) when many countries were under repeated strict social distances measures and when the information on the new virus was limited. It is likely that the mental health of children and adolescents changed with time.

In adults, studies on time trends in mental health during the pandemic have generally found that longer into the pandemic, the better their mental health, representing the psychological adaptation to the COVID-19 crisis [4–6]. In addition, the stringency of social distancing policies has been reported to show a strong negative association with mental well-being [7, 8]. However, this may not be generalizable to children and adolescents. Whereas many adults continued to work during lockdowns, albeit remotely, many youth experienced school closures, during which their lives changed completely (e.g., having no means of socializing with peers, being unable to participate in extracurricular activities, and spending much less time outside) [1]. In Japan, though national-level school closures were from April to May 2020, this was preceded by spring break in March 2020 and followed by summer vacation from July to August 2020, meaning that youth spent very little time in school during the first several months of the pandemic. Even as the school closures ended, many children and adolescents were asked to refrain from various activities that involved gathering and socializing due to social distancing policies in schools and restrictions in extracurricular activities. For instance, Japan’s Ministry of Education, Culture, Sports, Science and Technology has recommended limiting extracurricular activities to a minimum when the infection rates are high [9]. As extracurricular programs have been associated with improved mental well-being according to a previous study in pre-pandemic era [10], limiting school activities under social distancing policy may have been detrimental to the mental health of youth. Despite this, evidence on the associations between stringency of social distancing policy and mental well-being in children and adolescents is scarce.

Given their unique pandemic experience, the evaluation of the mental health of children and adolescents beyond the initial stages of the pandemic and a better understanding of its association with the stringency of the social distancing policies in place will have significant policy and clinical implications. Therefore, the present study aimed to describe long-term trends in emotional well-being and self-esteem among children and adolescents in Japan during the COVID-19 pandemic and its association with stringency of the social distancing policies in place. We also aimed to examine whether trends in these mental health outcomes differed by individual characteristics such as gender and age, in order to identify groups who are at highest risk of impaired mental health amidst the pandemic.

## Methods

### Study design

We investigated time trends in emotional well-being and self-esteem of children in Japan using the Corona-Codomo surveys, which collected serial cross-sectional data during the COVID-19 pandemic. These surveys explored the mental health and social experiences of youth aged 6–17 years of age and parents of youth aged 0–17 years and were repeated seven times in online format during the course of the pandemic: the first wave from April 30th to May 31st, 2020; the second wave from June 15th to July 31st, 2020; the third wave from September 1st to October 31st, 2020; the fourth wave from November 17th to December 27th, 2020; the fifth wave from February 19th to March 31st, 2021; the sixth wave from September 15th to September 30th, 2021; and the seventh from December 1st to December 31st, 2021. Participants were recruited through multiple platforms including the website of the National Center for Child Health and Development, child health organizations such as Japan Pediatric Society, social media, and other print and digital media platforms. The questionnaire consisted of self-report (age 6–17) and parent-report (age 0–17) questions, with slightly different questions depending on the age of the child. Children were able to participate on their own if their parent gave consent. The details of the study are described elsewhere [11, 12].

### Inclusion criteria

We included youth aged 6–17 years old whose self-reported answers to the question on the outcomes were available. We excluded participants with only parent-reported answers. Additionally, we excluded waves four and six of the Corona-Codomo survey since the outcome variables were not collected in these waves, and thus used data from waves 1, 2, 3, 5 and 7.

### Measures

#### Outcome variables: emotional well-being and self-esteem

Emotional well-being and self-esteem were measured using the self-report KINDL for children and adolescents, which is a well validated tool for measuring health-related quality of life in children and adolescents [13]. KINDL consists of six domains, physical, emotional, self-esteem, family, friends, and school. We focused on the emotional and self-esteem domains in the present study. Each domain is assessed using four questions, in which the participant chooses an answer from five categories (never, seldom, sometimes, often, or always) on quality of life in the past week. Each question is scored from 1 (never) to 5 (always), and a total is calculated for each domain. We transformed the scores into percentage scores, with a score of 100 representing the best quality of life in that domain (corresponding to a score of 5 for all four questions).

#### Explanatory variable: COVID-19 Stringency Index

As a measure of the stringency of social distancing policies, we utilized the COVID-19 Stringency Index by the Oxford COVID-19 government response tracker, which measures the stringency of containment and closure policy indicators for various countries [14]. The Index is computed using nine metrics of social distancing policy: school closures, workplace closures, cancellation of public events, restrictions on public gatherings, closures of public transport, stay-at-home requirements, public information campaigns, restrictions on internal movements, and international travel controls. The Index is calculated for each country on any given day, by taking the mean score of the nine metrics, taking a value between 0 and 100. Gender (male or female) and age group at baseline (6–11 or 12–17 years old) were used as covariates.

### Statistical analyses

#### Main analysis

We first conducted a descriptive analysis of participant characteristics for each wave. Second, we computed adjusted estimates for each outcome in each wave. Outcomes were adjusted with zero–one inflated beta (ZOIB) regression models to account for the non-normality of the outcomes and extreme scores of 0 and 100 [15]. The wave number was used as a categorical variable in all models. Adjusted models included interaction terms between the wave number and the gender or age group variable to take into account the varying sample characteristics in each wave. Then, we re-computed the estimates of the outcomes after weighting observations to reflect the distributions of age and gender in the Japanese population based on 2019 Census data (6–11 year-olds, 48.7%; 12–17 year-olds, 51.3%; males, 51.2%; and females, 48.8%) [16]. The weighted averages of the outcomes were superimposed on a timeline with the 7 day averages of COVID-19 cases [17], dates of school closures and academic breaks obtained from United Nations Educational, Scientific and Cultural Organization’s school closure dashboard [18], and dates of declarations of state of emergency. Finally, we tested the associations between Japan’s COVID-19 Stringency Index and mental health outcomes (emotional well-being and self-esteem). We created a ZOIB model for each outcome and plotted the adjusted estimates of the outcomes and 95% confidence intervals for each value of the Stringency Index in 5-unit increments from 0 to 100. We added additive three-way interaction terms between the Stringency Index and age group and gender, to see whether the associations between the Stringency Index and outcomes differed between subgroups. We created plots of the estimates with 95% confidence intervals (CIs) as well as plots stratified by age group and gender. Cluster-robust standard errors were used to take into account the dependencies between observations within each wave.

We performed all analysis with Stata SE version 15.1 (StataCorp, Texas, USA) and the command “margins.” 95% CIs were computed based on the delta method [19]. Two-sided P < 0.05 were considered statistically significant for all tests.

#### Missing data

Proportion of missing data for outcomes emotional well-being and self-esteem was 6.2% in wave 1, and zero in other waves (see flow chart in Additional file 1: Figure S1). The participant was allowed in the first wave to stop answering the questionnaire before reaching in the end, which may explain the missing data for the outcomes. Child’s age and gender were missing in 7.3%, 2.5%, 1.9%, 2.0%, and 1.2% of respondents in waves 1, 2, 3, 5, and 7, respectively. Adjusted analyses were performed on complete cases.

#### Sensitivity analyses

We conducted two sensitivity analyses. First, we excluded participants from three municipalities in the third survey, since data were collected using a different method in these municipalities (flyers with QR codes were provided to students through schools in addition to nationwide online recruitment). Next, we excluded individuals who participated in multiple surveys, who were identified based on their answer to a question in wave 3 (19.6% of participants replied that they had already participated in previous surveys). We performed this sensitivity analysis since individuals participating in multiple surveys could compromise the independence of the data across waves.

#### Ethical considerations

The study was approved by the institutional review board of the National Center for Child Health and Development (approval number 2020-21) and all parents gave informed consent to participate.

#### Role of the funding source

The Corona-Codomo survey was funded by the Ministry of Health, Labour and Welfare Grant (Grant Number 20GC1019; wave 1), Japan Science and Technology Agency J-RAPID Collaborative Research/Survey Program for Urgent Research on the Coronavirus Disease 2019 Grant (Grant Number JPMJJR2008; waves 2, 3, and 5), and Japan Science and Technology Agency SICORP Grant (wave 7). Analysis was conducted with funding by the Japan Society for the Promotion of Science JRP-LEAD with UKRI Grant (Grant Number JPJSJRP20211709).

## Results

The number of respondents aged 6–17 years old were 2430, 981, 2111, 501, and 487 in the first, second, third, fifth and seventh waves, respectively (Additional file 1: Figure S1). Table 1 reports the distributions of child age and gender for each wave. The 6- to 11-year-olds had a higher participation rate in waves 2 and 3 than in waves 1, 5, and 7. More females participated than males in all surveys ranging from 60.4% for wave 1–50.7% for wave 7. Other sociodemographic characteristics are shown in Additional file 1: Table S1.

**Table 1:** Basic characteristics and comparison between waves

	Wave 1		Wave 2		Wave 3		Wave 5		Wave 7	
	N = 2,252		N = 956		N = 2,070		N = 491		N = 481	
Child’s age	n	%	n	%	n	%	n	%	n	%
6–11	1,618	71.8	742	77.6	1,643	79.4	350	71.3	326	67.8
12–17	634	28.2	214	22.4	427	20.6	141	28.7	155	32.2
Child's gender	n	%	n	%	n	%	n	%	n	%
Male	860	38.2	424	44.4	850	41.1	199	40.5	234	48.6
Female	1,392	61.8	532	55.6	1220	58.9	292	59.5	247	51.4
Mental health outcomes	Mean	SD	Mean	SD	Mean	SD	Mean	SD	Mean	SD
Emotional well-being	72.6	20.2	74.4	20.4	76.3	19.3	72.6	23.0	72.9	20.6
Self-esteem	54.7	26.9	62.0	25.4	58.0	25.4	56.2	27.7	60.3	24.8

**Fig. 1:**
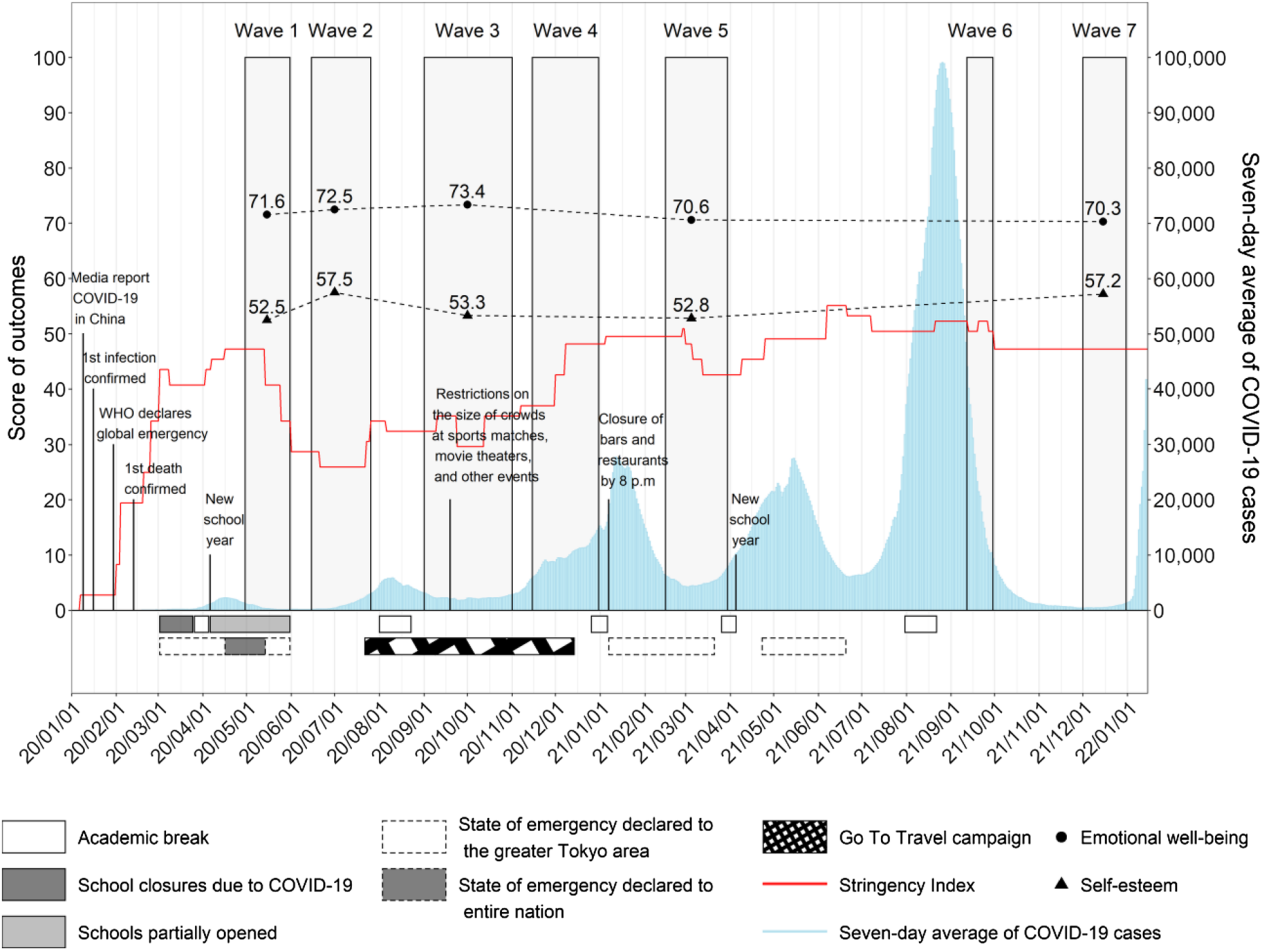
Trends in weighted adjusted (on age and gender) estimates of emotional well-being (dots) and self-esteem (triangles) superimposed on the 7 day average of the newly-reported COVID-19 cases, dates of school closures and academic breaks, the Stringency Index in Japan, and dates of declarations of state of emergency

Table 2 shows the weighted adjusted trends in emotional well-being and self-esteem over time among youth aged 6–17 years. Participants in May 2020 had the lowest self-esteem (adjusted mean, 52.5; 95% CI 51.3–53.7) compared to participants of other waves. Emotional well-being transiently increased from May 2020 to September/October 2020, but decreased into 2021; participants in December 2021 had the lowest emotional well-being (adjusted mean, 70.3; 95% CI 68.3–72.2). The results remained consistent after removing participants from three municipalities in the third wave and after removing individuals who participated in multiple surveys (Additional file 1: Tables S2 and S3). The increases in the weighted adjusted estimates of emotional well-being and self-esteem came after the school closures early in the pandemic in 2020. Although there were no school closures after this period, there were multiple declarations of emergencies over the course of the study period, in response to the several spikes in COVID-19 cases (Fig. 1).

**Table 2:** Weighted adjusted estimates of emotional well-being and self-esteem

	Estimate [95%CI]	Difference [95% CI]			
Emotional well-being					
May 2020	71.6 [70.7, 72.5]	reference			
July 2020	72.5 [70.9, 74.1]	0.9 [−1.0, 2.7], p = 0.350	reference		
Sep/Oct 2020	73.4 [72.3, 74.4]	1.8 [0.4, 3.2], p = 0.013	0.9 [−1.0, 2.8], p = 0.349	reference	
March 2021	70.6 [68.4, 72.8]	−1.0 [−3.4, 1.3], p = 0.401	−1.9 [−4.6, 0.8], p = 0.170	−2.8 [−5.2, −0.4], p = 0.024	reference
December 2021	70.3 [68.3, 72.2]	−1.4 [−3.5, 0.8], p = 0.213	−2.2 [−4.7, 0.3], p = 0.080	−3.1 [-5.3, −0.9], p = 0.005	−0.3 [−3.3, 2.6], p = 0.813
Self-esteem					
May 2020	52.5 [51.3, 53.7]	reference			
July 2020	57.5 [55.7, 59.4]	5.0 [2.8, 7.3], p < 0.001	reference		
Sep/Oct 2020	53.3 [52.0, 54.6]	0.8 [−0.9, 2.6], p = 0.353	−4.2 [−6.5, −1.9], p < 0.001	reference	
March 2021	52.8 [50.2, 55.5]	0.3 [−2.6, 3.2], p = 0.831	−4.7 [−8.0, −1.5], p = 0.004	−0.5 [−3.5, 2.4], p = 0.726	reference
December 2021	57.2 [54.9, 59.4]	4.7 [2.1, 7.3], p < 0.001	−0.3 [−3.3, 2.6], p = 0.821	3.9 [1.3, 6.5], p = 0.004	4.4 [0.9, 7.9], p = 0.014

**Fig. 2:**
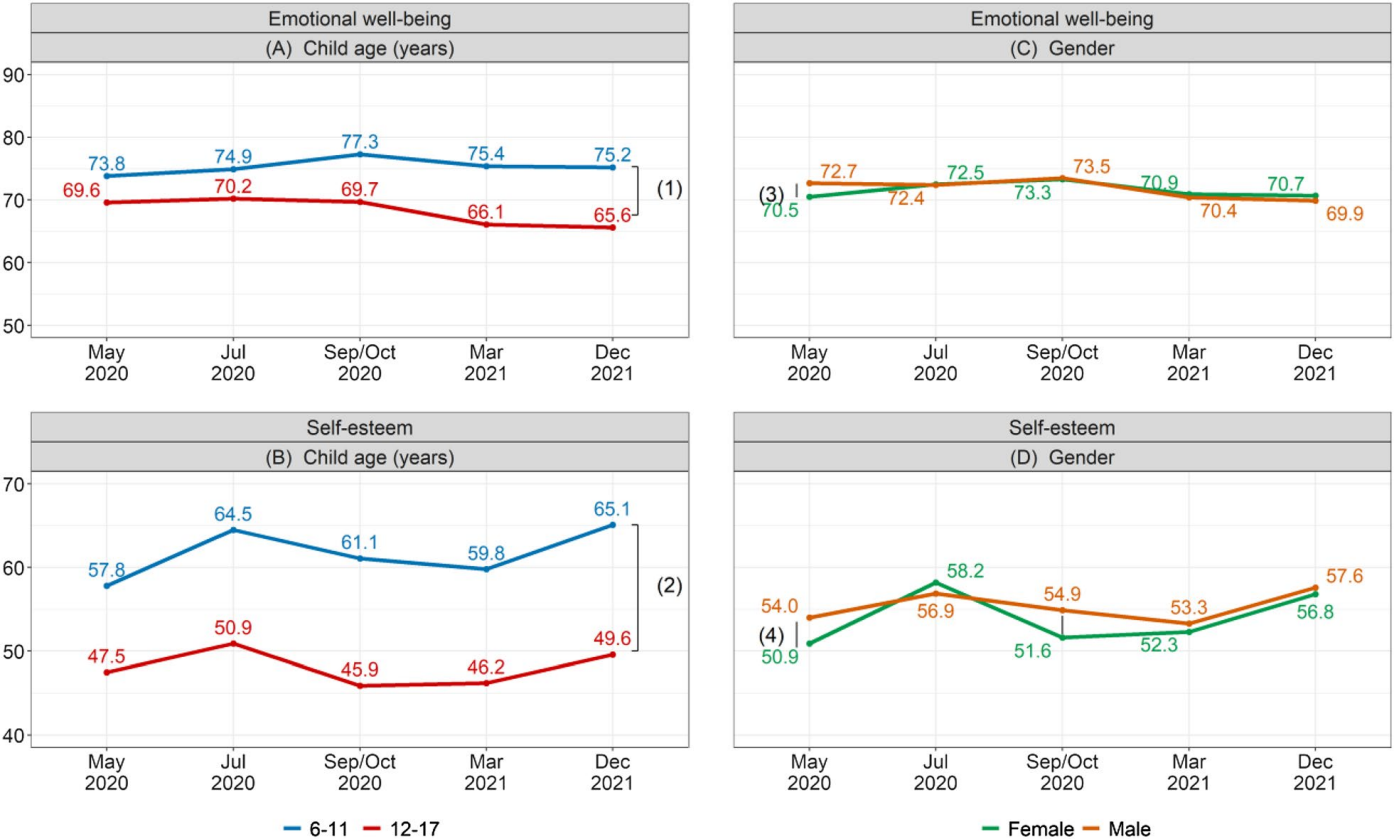
Weighted adjusted estimates of emotional well-being and self-esteem by age and gender. (1) Differences in emotional well-being between 6- to 11-year-olds and 12- to 17-year-olds were statistically significant in all waves (p < 0.005). (2) Differences in self-esteem between 6- to 11-year-olds and 12- to 17-year-olds were statistically significant in all waves (p < 0.001). (3) The difference in emotional well-being between females and males was statistically significant in May 2020 only (p = 0.006). (4) Differences in self-esteem between females and males were statistically significant in May 2020 (p = 0.003) and Sep/Oct 2021 (p = 0.001). See Additional file 1: Tables S4 and S5 for comparisons between waves

Participants aged 12–17 years had lower emotional well-being and self-esteem compared to 6- to 11-year-olds in all waves (Fig. 2A and B, Additional file 1: Table S4). Females had lower emotional well-being than males in May 2020 (70.5 for females vs 72.7 for males; p = 0.006; Fig. 2C, Additional file 1: Table S5) and lower self-esteem than males in May 2020 (50.9 for females vs 54.0 for males; p = 0.003; Fig. 2D, Additional file 1: Table S5) and September/October 2020 (51.6 for females vs 54.9 for males; p = 0.001; Fig. 2D, Additional file 1: Table S5).

**Fig. 3:**
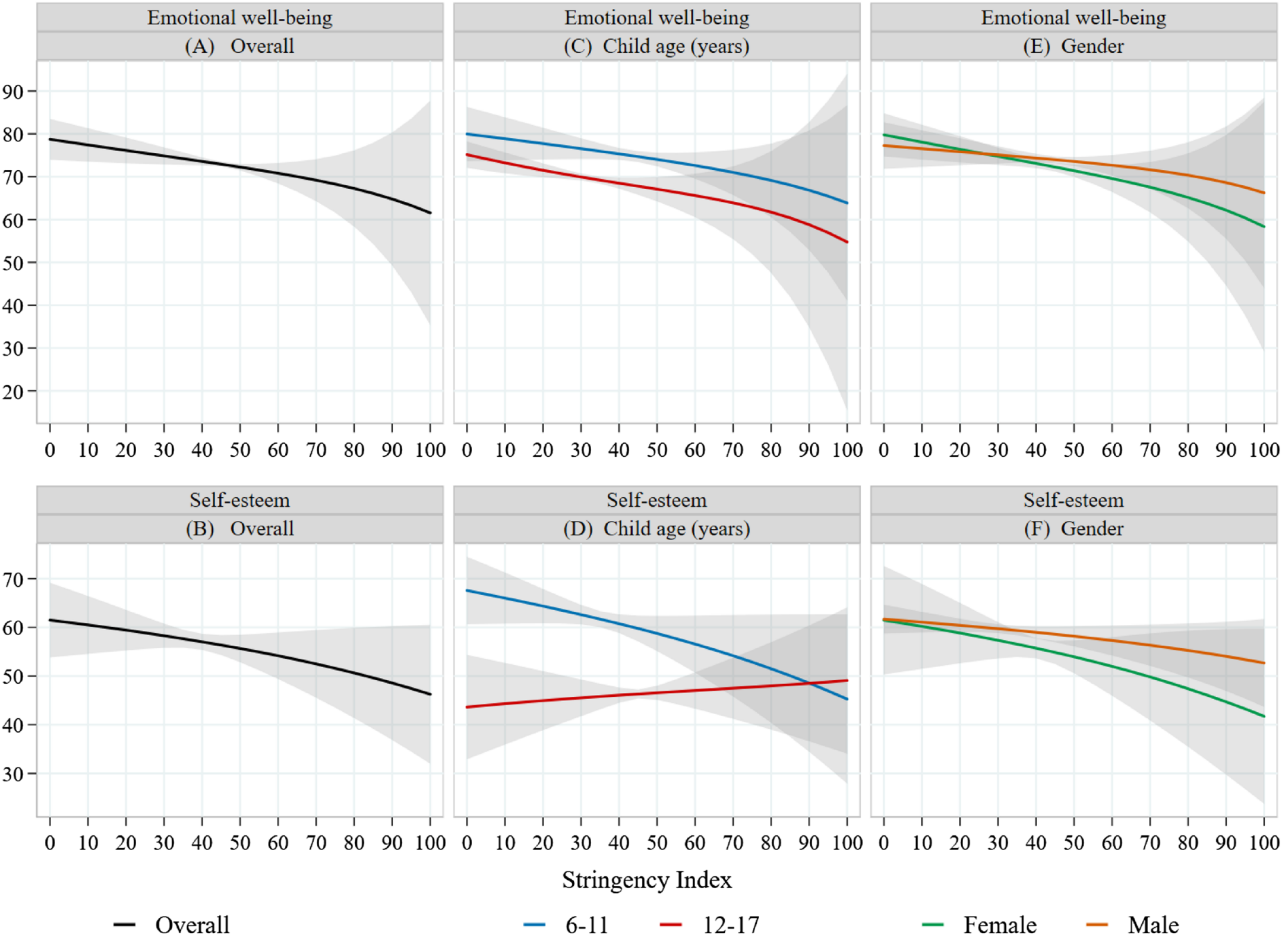
Relationship between the Stringency Index and adjusted estimates of emotional well-being and self-esteem in the full sample and by age and gender (see Additional file 1: Table S5 for details). A zero–one inflated beta regression model was created for each outcome and adjusted estimates of the outcomes and 95% confidence intervals were plotted for each value of the Stringency Index in 5-unit increments from 0 to 100

In general, there was a negative association with stringency of social distancing policies for both emotional well-being (coefficient, −1.30; 95% CI −2.55 to −0.05; Fig. 3A, Additional file 1: Table S6) and self-esteem (coefficient, −1.29; 95% CI −3.32 to 0.74; Fig. 3B, Additional file 1: Table S6) in absolute scales for a 5-unit increase in the Stringency Index, though the estimate for self-esteem was imprecise with a large CI. As the observed Stringency Indices ranged from 25.9 to 50.9, the confidence intervals of some of the estimates are large and the estimates of the outcomes for Stringency Indices outside the observed range are pure predictions. We did not find evidence that the association between stringency of social distancing policies and emotional well-being differed by age group (Fig. 3C, Additional file 1: Table S6), but children aged 6–11 had a more negative association between Stringency Index and self-esteem than those aged 12–17 (coefficient for interaction term with 12- to 17-year-olds as reference, −2.44; 95% CI −4.49 to −0.39; Fig. 3D, Additional file 1: Table S6). There was a negative association between Stringency Index and emotional well-being for females (coefficient, −1.67; 95% CI −3.11 to −0.24; Fig. 3E, Additional file 1: Table S6), but this was not statistically significant for males. We did not find a statistically significant association between Stringency Index and self-esteem for either gender (Fig. 3F, Additional file 1: Table S6).

## Discussion

We found that emotional well-being and self-esteem of children and adolescents in Japan improved transiently in the few months from the start of the pandemic to Summer to Autumn 2020, followed by a slight worsening trend into the second year of the pandemic. While emotional well-being stayed lower compared to initial levels nearly 2 years into the pandemic, self-esteem began to improve by late 2021. Our subgroup analyses revealed that emotional well-being and self-esteem were lower for adolescents compared to younger children aged 6–11 years throughout the course of the pandemic, but gender differences were small. Children and adolescents exposed to more stringent social distancing measures tended to have lower emotional well-being and self-esteem in general. However, the association for stringency and self-esteem differed by age group: children aged 6–11 years tended to have lower self-esteem with more stringent measures, while those aged 12–17 years did not display this trend. Furthermore, emotional well-being among females tended to be particularly lower with more stringent measures, although there was no statistically significant gender difference.

The overall trends in emotional well-being and self-esteem mimic that of mental health after disasters: the affected people’s mental health is known to improve transiently immediately after a disaster, then follows a worsening pattern. This is termed the “honeymoon effect,” in which factors like the collective feelings of concern and altruism are thought to prevent the worsening of mental health in the initial phases after an event, and demoralization, anxiety, and lifestyle changes set in in later phases [20]. For instance, after the Great Eastern Japan Earthquake in 2011, suicide death rates in Fukushima, Japan declined in the first 2 years after the disaster but surpassed pre-disaster levels in the third year [21]. While it is typical that this initial improving-worsening pattern occurs over a span of several years, the unprecedented and ongoing nature of the COVID-19 pandemic may have caused it to happen over a span of months. This is further corroborated by the increase in suicide rates in Japan following an initial decline during the COVID-19 pandemic [22], which mirrors the trends in self-esteem in our study sample. Interestingly, in contrast to a previous study that suggested that the detrimental effects of war on the emotional development of young children worsened with chronic exposure [23], the self-esteem of children in December 2021 showed an improving pattern in our study. This is likely multifactorial, but it may be that children and the society started to get used to the pandemic and the social distancing measures, 2 years into the pandemic (whereas it is much more difficult to rebuild society when it is chronically vulnerable to the destructions of war). Furthermore, we found that self-esteem began to improve later in 2021, while emotional well-being did not. A previous study has suggested the mediating role of self-esteem in the relationship between stress and psychological well-being [24], and our findings may reflect this; with improvement in self-esteem, well-being may subsequently improve. To get a clearer picture of the long-term consequences of the pandemic, future studies should track the longitudinal changes in the self-esteem and emotional well-being of children affected by the pandemic over a longer period of time.

Generally, we found that the stringency of social distancing policies was negatively associated with children and adolescents’ emotional well-being and self-esteem, which is in line with previous studies that showed a negative association between the Stringency Index and mental health among adults [7, 8] and young children aged 3–8 years [25]. Our study expands the findings from these previous studies by showing that a negative association exists between the stringency of social distancing policies and self-reported emotional well-being and self-esteem of both children and adolescents. In line with the argument by many experts that school closures and social distancing policies during the pandemic put massive strain on children and adolescents [26, 27], our study suggests that stringent social distancing policies should be implemented with caution to protect the mental health of children and adolescents. Indeed, as evidence suggests that the effects of some social distancing measures (e.g., school closures) in containing COVID-19 may be limited in some settings (although at the time of the study, there were extremely few pediatric COVID-19 cases, and as the authors suggest, it is possible that school closures could have a discernible effect on COVID-19 infections once the reproduction number within a school environment is greater than a certain threshold) [28], policymakers should consider the risks and benefits of implementing such policies, especially from the perspective of children and adolescents. In the context of youth in Japan, the high stringency index could reflect school closures and restrictions in various school-related activities. For instance, children and adolescents spent a significant amount of time away from school early in the pandemic, with spring break in March 2020, national-level school closures from April to May 2020, and summer vacation from July to August 2020. Later in the pandemic, students were still asked to refrain from gathering and socializing at schools and extracurricular activities [9]. Possibly reflecting this, suicides attributed to social concerns increased in Japan from summer 2020, underscoring the importance of regular social interactions [29]. To protect the mental well-being of youth during a pandemic, opportunities for regular social interactions may be beneficial.

The age group and gender differences in emotional well-being and self-esteem found in our study agree with previous studies conducted before the pandemic, which suggest that subjective well-being decreases with age during adolescence [30] and that females tend to report lower subjective well-being than males [31]. The finding that younger and female children may have been especially vulnerable to stringent social distancing policy is, to our knowledge, novel. Younger children may have been especially affected by social distancing policy since they had fewer means to communicate with their peers during school closures and lockdowns: younger children spend less time on social media [32] and fewer of them own mobile phones [33], meaning they are less likely to have access to the primary means of communicating with peers during lockdowns. Furthermore, our finding that females appeared to be more vulnerable to the stringency of closure policy is not unexpected. A previous study has shown that during the COVID-19 pandemic, women reported lower self-esteem while social distancing compared to men [34]. The authors suggested that this relationship may be mediated by the higher need to belong, which may explain our findings too. Analyses of factors mediating the relationship between the stringency of social distancing policies and the changes in mental health are warranted to explore this further.

### Limitations

Our study should be interpreted in light of several limitations. First, the study samples used in each wave could be heterogeneous, as participants were recruited in each wave, and may not necessarily represent the Japanese population. To account for this limitation, we produced estimates adjusted to reflect the distributions of age and gender in the Japanese population, although we could not take into account differences by location. Future studies should use a longitudinal, nationally-representative sample to produce more accurate estimates of the national trend in the mental health of children in Japan.

Our analyses of the relationship between stringency of social distancing policy and the outcomes should be interpreted with caution, as the COVID-19 Stringency Index is comprised of several policies, including school closures, closure of workplaces, and stay-at-home orders [14]. Different nonpharmaceutical interventions are hypothesized to affect children heterogeneously, and this may not necessarily be reflected in the index. For instance, school closures were only imposed in the early phases of the pandemic, but the Stringency Index is higher in the later phases of the pandemic. In addition, there may have been unmeasured confounding between the Stringency Index and the outcomes, and the associations explored in this study do not necessarily represent causal relationships.

## Conclusion

During the COVID-19 pandemic, the emotional well-being and self-esteem of children and adolescents in Japan improved transiently from the start of the pandemic to Summer to Autumn 2020, followed by a slight worsening trend into the second year of the pandemic. While emotional well-being stayed lower compared to initial levels nearly 2 years into the pandemic, self-esteem began to improve by late 2021. Our finding that more stringent social distancing measures was associated with lower emotional well-being and self-esteem, especially 6–11 year-olds’ self-esteem and females’ emotional well-being, highlights the need to further examine the impact of social distancing measures on children and adolescents’ mental health, with a focus on vulnerable populations.

## Supplementary Information

**Additional file 1: Figure S1. **Sample selection procedure. **Table S1.** Sociodemographic characteristics. **Table S2.** Weighted adjusted estimates of emotional well-being and self-esteem, excluding three municipalities participating in wave 3. **Table S3.** Weighted adjusted estimates of emotional well-being and self-esteem, excluding participants who reported having answered previous surveys (information only available in wave 3). **Table S4.** Weighted adjusted estimates and differences in emotional well-being and self-esteem, stratified by age (Fig. 2). **Table S5.** Weighted adjusted estimates and differences in emotional well-being and self-esteem stratified by gender (Fig. 2). **Table S6.** Associations between the Stringency Index and mental health outcomes and coefficients of multiplicative interaction terms between the Stringency Index and age group and gender.

## Data Availability

Aggregate data of the Corona-Codomo study can be shared upon request. Individual data can only be shared between research collaborators.
