# Supplementary material for: Time trends in emotional well-being and self-esteem in children and adolescents during the COVID-19 pandemic
Source: Child Adolesc Psychiatry Ment Health. 2022 Nov 23;16:89. doi: 10.1186/s13034-022-00525-3 (PMC9685852; doi:10.1186/s13034-022-00525-3)
Supplement: Supplementary file 1 — Additional file 1: Figure S1. Sample selection procedure. Table S1. Sociodemographic characteristics. Table S2. Weighted adjusted estimates of emotional well-being and self-esteem, excluding three municipalities participating in wave 3. Table S3. Weighted adjusted estimates of emotional well-being and self-esteem, excluding participants who reported having answered previous surveys (information only available in wave 3). Table S4. Weighted adjusted estimates and differences in emotional well-being and self-esteem, stratified by age (Fig. 2). Table S5. Weighted adjusted estimates and differences in emotional well-being and self-esteem stratified by gender (Fig. 2). Table S6. Associations between the Stringency Index and mental health outcomes and coefficients of multiplicative interaction terms between the Stringency Index and age group and gender. [file 13034_2022_525_MOESM1_ESM.pdf]

Time trends in emotional well-being and self-esteem in children and adolescents during the COVID-19 pandemic

Supplemental Material

Figure S1. Sample selection procedure. 2

Table S1. Sociodemographic characteristics. 3

Table S2. Weighted adjusted estimates of emotional well-being and self-esteem, excluding three municipalities participating in wave 3. 4

Table S3. Weighted adjusted estimates of emotional well-being and self-esteem, excluding participants who reported having answered previous surveys (information only available in wave 3). 5

Table S4. Weighted adjusted estimates and differences in emotional well-being and self-esteem, stratified by age (Figure 2). 6

Table S5. Weighted adjusted estimates and differences in emotional well-being and self-esteem stratified by gender (Figure 2). 7

Table S6. Associations between the Stringency Index and mental health outcomes and coefficients of multiplicative interaction terms between the Stringency Index and age group and gender. 8

Figure S1. Sample selection procedure.

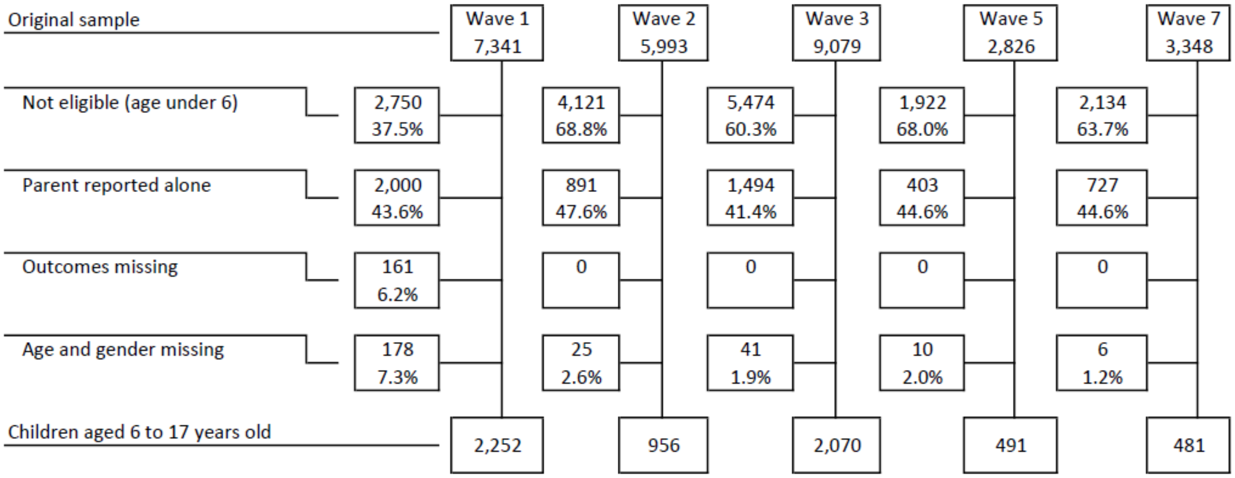

Table S1. Sociodemographic characteristics.

	Wave 1 N= 2,252		Wave 2 N= 956		Wave 3 N= 2,070		Wave 5 N= 491		Wave 7 N= 481	
Who the child lives with										
	n	%	n	%	n	%	n	%	n	%
Living with both parents	1966	87.3					406	82.7	399	83.0
Living with one parent/others	273	12.1					80	16.3	82	17.0
Siblings										
No	637	28.3					169	34.4	151	31.4
Yes	1610	71.5					318	64.8	330	68.6
Subjective Household Financial Status*										
	n	%	n	%	n	%	n	%	n	%
Okay			766	80.1	1525	73.7	361	73.5	379	78.8
Somewhat/very difficult			164	17.2	482	23.3	116	23.6	97	20.2
Subjective financial status compared to 2020*										
	Mean	SD	Mean	SD	Mean	SD	Mean	SD	Mean	SD
Better now	38	3.3	39	4.1	90	4.3	22	4.5	27	5.6
Same	829	71.6	685	71.7	1380	66.7	317	64.6	342	71.1
Worse now	274	23.7	202	21.1	491	23.7	124	25.3	103	21.4

*Not asked when child participated alone in wave 1

Table S2. Weighted adjusted estimates of emotional well-being and self-esteem, excluding three municipalities participating in wave 3.

	N	Estimate [95%CI]	Difference [95% CI]			
Emotional well-being						
May 2020	2,252	71.6 [70.7, 72.5]	reference			
July 2020	956	72.5 [70.9, 74.1]	0.9 [-0.9, 2.7], p=0.350	reference		
Sep/Oct 2020	1,545	73.9 [72.7, 75.0]	2.2 [0.8, 3.7], p=0.003	1.4 [-0.6, 3.3], p=0.168	reference	
March 2021	491	70.6 [68.4, 72.8]	-1.0 [-3.4, 1.3], p=0.401	-1.9 [-4.6, 0.8], p=0.170	-3.2 [-5.7, -0.8], p=0.010	reference
December 2021	481	70.3 [68.3, 72.2]	-1.4 [-3.5, 0.8], p=0.213	-2.2 [-4.7, 0.3], p=0.080	-3.6 [-5.9, -1.3], p=0.002	-0.4 [-3.3, 2.6], p=0.813
Self-esteem						
May 2020	2,252	52.5 [51.3, 53.7]	reference			
July 2020	956	57.5 [55.7, 59.4]	5.0 [2.8, 7.3], p<0.001	reference		
Sep/Oct 2020	1,545	53.5 [52.1, 54.9]	1.0 [-0.9, 2.9], p=0.289	-4.0 [-6.4, -1.7], p=0.001	reference	
March 2021	491	52.8 [50.2, 55.5]	0.3 [-2.6, 3.2], p=0.831	-4.7 [-8.0, -1.5], p=0.004	-0.7 [-3.7, 2.3], p=0.649	reference
December 2021	481	57.2 [54.9, 59.4]	4.7 [2.1, 7.3], p<0.001	-0.3 [-3.3, 2.6], p=0.821	3.7 [1.0, 6.3], p=0.007	4.4 [0.9, 7.9], p=0.014

Table S3. Weighted adjusted estimates of emotional well-being and self-esteem, excluding participants who reported having answered previous surveys (information only available in wave 3).

	N	Estimate [95%CI]	Difference [95% CI]			
Emotional well-being						
May 2020	2,252	71.6 [70.7, 72.5]	reference			
July 2020	956	72.5 [70.9, 74.0]	0.9 [-0.9, 2.7], p=0.350	reference		
Sep/Oct 2020	1,691	72.7 [71.5, 73.8]	1.1 [-0.4, 2.5], p=0.156	0.2 [-1.7, 2.1], p=0.843	reference	
March 2021	491	70.6 [68.4, 72.8]	-1.0 [-3.4, 1.3], p=0.402	-1.9 [-4.6, 0.8], p=0.171	-2.1 [-4.5, 0.4], p=0.099	reference
December 2021	481	70.2 [68.3, 72.2]	-1.4 [-3.5, 0.8], p=0.213	-2.2 [-4.7, 0.3], p=0.080	-2.4 [-4.7, -0.2], p=0.035	-0.4 [-3.3, 2.6], p=0.813
Self-esteem						
May 2020	2,252	52.5 [51.3, 53.7]	reference			
July 2020	956	57.5 [55.7, 59.4]	5.0 [2.8, 7.3], p<0.001	reference		
Sep/Oct 2020	1,691	52.7 [51.2, 54.1]	0.2 [-1.7, 2.1], p=0.845	-4.8 [-7.2, -2.5], p<0.001	reference	
March 2021	491	52.8 [50.2, 55.5]	0.3 [-2.6, 3.2], p=0.831	-4.7 [-8.0, -1.5], p=0.004	0.1 [-2.9, 3.1], p=0.933	reference
December 2021	481	57.2 [54.9, 59.4]	4.7 [2.1, 7.3], p<0.001	-0.3 [-3.3, 2.6], p=0.821	4.5 [1.8, 7.2], p=0.001	4.4 [0.9, 7.9], p=0.014

Table S4. Weighted adjusted estimates and differences in emotional well-being and self-esteem, stratified by age (Figure 2).

	Estimate [95%CI]	Difference [95% CI]			
**Emotional well-being**					
6-11					
May 2020	73.8 [72.9, 74.7]	reference			
July 2020	74.9 [73.5, 76.3]	1.1 [-0.6, 2.7], p=0.194	reference		
Sep/Oct 2020	77.3 [76.4, 78.2]	3.4 [2.2, 4.7], p<0.001	2.4 [0.7, 4.0], p=0.004	reference	
March 2021	75.4 [73.2, 77.5]	1.6 [-0.8, 3.9], p=0.188	0.5 [-2.1, 3.0], p=0.715	-1.9 [-4.2, 0.4], p=0.110	reference
December 2021	75.2 [73.1, 77.2]	1.3 [-0.9, 3.6], p=0.248	0.2 [-2.2, 2.7], p=0.845	-2.1 [-4.4, 0.1], p=0.067	-0.2 [-3.2, 2.7], p=0.882
12-17					
May 2020	69.6 [68.0, 71.1]	reference			
July 2020	70.2 [67.5, 73.0]	0.7 [-2.5, 3.8], p=0.683	reference		
Sep/Oct 2020	69.7 [67.9, 71.6]	0.2 [-2.2, 2.6], p=0.883	-0.5 [-3.8, 2.8], p=0.778	reference	
March 2021	66.1 [62.5, 69.7]	-3.5 [-7.4, 0.5], p=0.086	-4.1 [-8.7, 0.5], p=0.078	-3.6 [-7.7, 0.4], p=0.081	reference
December 2021	65.6 [62.4, 68.8]	-3.9 [-7.5, -0.4], p=0.030	-4.6 [-8.8, -0.4], p=0.033	-4.1 [-7.8, -0.4], p=0.029	-0.5 [-5.3, 4.4], p=0.848
**Self-esteem**					
6-11					
May 2020	57.8 [56.5, 59.1]	reference			
July 2020	64.5 [62.8, 66.3]	6.8 [4.6, 8.9], p<0.001	reference		
Sep/Oct 2020	61.1 [59.9, 62.3]	3.3 [1.6, 5.1], p<0.001	-3.4 [-5.5, -1.3], p=0.002	reference	
March 2021	59.8 [57.0, 62.5]	2.0 [-1.0, 5.0], p=0.199	-4.8 [-8.0, -1.5], p=0.004	-1.4 [-4.4, 1.6], p=0.370	reference
December 2021	65.1 [62.6, 67.6]	7.4 [4.6, 10.2], p<0.001	0.6 [-2.4, 3.6], p=0.699	4.0 [1.3, 6.8], p=0.004	5.4 [1.7, 9.1], p=0.004
12-17					
May 2020	47.5 [45.5, 49.4]	reference			
July 2020	50.9 [47.7, 54.0]	3.4 [-0.3, 7.1], p=0.072	reference		
Sep/Oct 2020	45.9 [43.7, 48.1]	-1.5 [-4.5, 1.4], p=0.306	-4.9 [-8.8, -1.1], p=0.012	reference	
March 2021	46.2 [42.0, 50.4]	-1.3 [-5.9, 3.4], p=0.594	-4.7 [-9.9, 0.6], p=0.082	0.3 [-4.5, 5.0], p=0.912	reference
December 2021	49.6 [46.1, 53.2]	2.2 [-1.9, 6.2], p=0.292	-1.2 [-6.0, 3.5], p=0.612	3.7 [-0.5, 7.9], p=0.082	3.4 [-2.1, 8.9], p=0.221

Table S5. Weighted adjusted estimates and differences in emotional well-being and self-esteem stratified by gender (Figure 2).

	Estimate [95%CI]	Difference [95% CI]			
Emotional well-being					
Male					
May 2020	72.7 [71.4, 74.0]	reference			
July 2020	72.4 [70.4, 74.5]	-0.3 [-2.7, 2.1], p=0.822	reference		
Sep/Oct 2020	73.5 [72.1, 74.9]	0.8 [-1.2, 2.7], p=0.441	1.0 [-1.4, 3.5], p=0.413	reference	
March 2021	70.4 [67.2, 73.5]	-2.4 [-5.8, 1.1], p=0.175	-2.1 [-5.8, 1.7], p=0.277	-3.1 [-6.6, 0.3], p=0.077	reference
December 2021	69.9 [67.3, 72.5]	-2.9 [-5.8, 0.0], p=0.054	-2.6 [-5.9, 0.7], p=0.125	-3.6 [-6.6, -0.7], p=0.016	-0.5 [-4.6, 3.6], p=0.811
Female					
May 2020	70.5 [69.4, 71.6]	reference			
July 2020	72.5 [70.6, 74.5]	2.1 [-0.2, 4.3], p=0.071	reference		
Sep/Oct 2020	73.3 [72.1, 74.6]	2.8 [1.2, 4.5], p=0.001	0.8 [-1.6, 3.1], p=0.518	reference	
March 2021	70.9 [68.4, 73.4]	0.4 [-2.3, 3.2], p=0.772	-1.7 [-4.9, 1.5], p=0.310	-2.4 [-5.2, 0.4], p=0.091	reference
December 2021	70.7 [68.1, 73.3]	0.2 [-2.6, 3.0], p=0.886	-1.9 [-5.1, 1.4], p=0.268	-2.6 [-5.5, 0.3], p=0.077	-0.2 [-3.8, 3.5], p=0.915
Self-esteem					
Male					
May 2020	54.0 [52.3, 55.7]	reference			
July 2020	56.9 [54.5, 59.3]	2.9 [-0.1, 5.9], p=0.055	reference		
Sep/Oct 2020	54.9 [53.2, 56.7]	0.9 [-1.5, 3.4], p=0.451	-2.0 [-4.9, 1.0], p=0.194	reference	
March 2021	53.3 [49.6, 57.0]	-0.7 [-4.8, 3.4], p=0.743	-3.6 [-8.0, 0.8], p=0.110	-1.6 [-5.7, 2.4], p=0.434	reference
December 2021	57.6 [54.7, 60.5]	3.6 [0.2, 7.0], p=0.039	0.7 [-3.1, 4.5], p=0.724	2.7 [-0.8, 6.1], p=0.127	4.3 [-0.4, 9.0], p=0.075
Female					
May 2020	50.9 [49.5, 52.3]	reference			
July 2020	58.2 [55.9, 60.5]	7.3 [4.6, 10.0], p<0.001	reference		
Sep/Oct 2020	51.6 [50.2, 53.1]	0.7 [-1.3, 2.8], p=0.475	-6.5 [-9.3, -3.8], p<0.001	reference	
March 2021	52.3 [49.2, 55.3]	1.4 [-2.0, 4.7], p=0.428	-5.9 [-9.7, -2.1], p=0.003	0.6 [-2.8, 4.0], p=0.719	reference
December 2021	56.8 [53.8, 59.8]	5.9 [2.6, 9.2], p=0.001	-1.4 [-5.2, 2.4], p=0.463	5.1 [1.8, 8.4], p=0.003	4.5 [0.2, 8.8], p=0.040

Table S6. Associations between the Stringency Index and mental health outcomes and coefficients of multiplicative interaction terms between the Stringency Index and age group and gender.

	Coefficient	95%CI		p-value
Emotional well-being				
Overall	-1.30	-2.55	-0.05	0.041
Child Age				
6-11	-1.26	-2.81	0.29	0.111
12-17	-1.44	-3.01	0.13	0.072
Interaction	0.18	-1.94	2.30	0.866
Gender				
Female	-1.67	-3.11	-0.24	0.022
Male	-0.77	-2.06	0.52	0.241
Interaction	-0.60	-1.81	0.60	0.324
6-11/Female	-1.62	-3.61	0.36	0.109
6-11/Male	-0.74	-1.98	0.51	0.245
12-17/Female	-1.84	-3.49	-0.19	0.029
12-17/Male	-0.87	-2.86	1.11	0.389
Self-esteem				
Overall	-1.29	-3.32	0.74	0.212
Child Age				
6-11	-1.92	-4.04	0.20	0.076
12-17	0.52	-1.88	2.93	0.671
Interaction	-2.44	-4.49	-0.39	0.020
Gender				
Female	-1.68	-4.49	1.14	0.243
Male	-0.76	-1.73	0.20	0.122
Interaction	-0.60	-1.34	0.15	0.118
6-11/Female	-2.42	-5.06	0.21	0.072
6-11/Male	-1.19	-2.62	0.24	0.104
12-17/Female	0.54	-3.61	4.70	0.798
12-17/Male	0.49	-0.27	1.26	0.209

The coefficients were calculated for 10-unit increases in the Stringency Index, for the full sample and stratified by age group and gender (Figure 3).
